# Incidence and prediction of intraoperative and postoperative cardiac arrest requiring cardiopulmonary resuscitation and 30-day mortality in non-cardiac surgical patients

**DOI:** 10.1371/journal.pone.0225939

**Published:** 2020-01-22

**Authors:** Heiko A. Kaiser, Nahel N. Saied, Andreas S. Kokoefer, Lina Saffour, Jonathan K. Zoller, Mohammad A. Helwani

**Affiliations:** 1 Department of Anesthesiology, Washington University, St. Louis, Missouri, United States of America; 2 Department of Anesthesiology and Pain Medicine; Inselspital, Bern University Hospital; University of Bern, Freiburgstrasse, Bern, Switzerland; 3 Department of Anesthesiology and Critical Care, University of Arkansas Medical Sciences, Little Rock, Arkansas, United States of America; 4 Department of Anesthesiology, Perioperative Care and Intensive Care Medicine, Paracelsus Medical University Salzburg, Strubergasse, Salzburg, Austria; Azienda Ospedaliero Universitaria Careggi, ITALY

## Abstract

**Background:**

The incidence, prediction and mortality outcomes of intraoperative and postoperative cardiac arrest requiring cardiopulmonary resuscitation (CPR) in surgical patients are under investigated and have not been studied concurrently in a single study.

**Methods:**

A retrospective cohort study was conducted using the American College of Surgeons National Surgical Quality Improvement Program data between 2008 and 2012. Firth’s penalized logistic regression was used to study the incidence and identify risk factors for intra- and postoperative CPR and 30-day mortality. simplified prediction model was constructed and internally validated to predict the studied outcomes.

**Results:**

Among about 1.86 million non-cardiac operations, the incidence rate of intraoperative CPR was 0.03%, and for postoperative CPR was 0.33%. The 30-day mortality incidence rate was 1.25%. The incidence rate of events decreased overtime between 2008–2012. Of the 29 potential predictors, 14 were significant for intraoperative CPR, 23 for postoperative CPR, and 25 for 30-day mortality. The five strongest predictors (highest odd ratios) of intraoperative CPR were the American Society of Anesthesiologists (ASA) physical status, Systemic Inflammatory Response Syndrome (SIRS)/sepsis, surgery type, urgent/emergency case and anesthesia technique. Intraoperative CPR, ASA, age, functional status and end stage renal disease were the most significant predictors for postoperative CPR. The most significant predictors of 30-day mortality were ASA, age, functional status, SIRS/sepsis, and disseminated cancer. The predictions with the simplified five-factor model performed well and was comparable to the full prediction model. Postoperative cardiac arrest requiring CPR, compared to intraoperative, was associated with much higher mortality.

**Conclusions:**

The incidence of cardiac arrest requiring CPR in surgical patients decreased overtime. Risk factors for intraoperative CPR, postoperative CPR and perioperative mortality are overlapped. We proposed a simplified approach compromised of five-factor model to identify patients at high risk. Postoperative, compare to intraoperative, cardiac arrest requiring CPR was associated with much higher mortality.

## Introduction

In-hospital cardiopulmonary resuscitation (CPR) is a rare but devastating event. It is estimated that there are at least 200,000 cases of treated in- hospital cardiac arrests requiring Advance Cardiac Life Support per year in US hospitals.[1] Among these cases, there is a well-defined subgroup of patients (surgical patients) who require CPR in the perioperative period. Between 2005 and 2010, nearly one in 200 surgical patients underwent CPR. [2] Among these patients, three-quarters suffered from a postoperative complication before or on the day of CPR, and more than two thirds of them died in the first 30 days after surgery.[2] Every incident of cardiac arrest requiring CPR is not only harmful to the patient, but it is also taxing on the hospital staff, the patients’ families, and adds additional financial burden to the health care system. For aforesaid reasons, identifying and reducing patient-specific risk factors is imperative to improve both patient safety and hospital cost.

In an attempt to improve surgical patient outcomes, the American College of Surgeons (ACS) developed the National Surgical Quality Improvement Program (NSQIP). This program collects patient-specific variables and 30-day postoperative occurrences specifically for the surgical patient population. While NSQIP has developed a surgical risk calculator for 30-day postoperative complications and death, the calculator incorporates multiple variables, and can be cumbersome, time consuming, and difficult to integrate in the daily busy clinical practice as it requires the surgeon to enter 22 preoperative patient risk factors about their patients (https://Riskcalculator.Facs.org).

The purpose of this study is to investigate both the incidence of intraoperative and 30-day postoperative cardiac arrest requiring CPR and mortality in patients who underwent perioperative CPR. Also, we aimed to identify simplified model for cardiac arrest requiring CPR and 30-day mortality in a disaggregate manner. This simplified risk assessment approach might be of great utility in the busy daily practice for health care providers to quickly and easily estimate the risk of cardiac arrest requiring CPR and mortality in surgical patients. This estimate, with optimizing risk factors, may mobilize resources, increase monitoring and guide selection of surgical interventions to minimize these serious complications.

## Materials and methods

### Data source

We used data from the ACS-NSQIP from January 1, 2008 through December 31, 2012. The year 2012 was chosen as the endpoint because after 2012 the NSQIP database stopped collecting data on intraoperative CPR. The ACS NSQIP is conducted under institutional review board approval at Barnes-Jewish Hospital, Washington University in St. Louis. The present study was performed with use of the preexisting and deidentified Participant Use Data File and thus was exempt from further review. The STROBE checklist for observational studies was used to guide the methods of this study and to structure this manuscript.[3]

### Inclusion and exclusion criteria

All patients over the age of 18 undergoing non-cardiac surgery were included, except for oral and eye surgeries due to low count in the database. Patients with missing data in one or more variables of interest were excluded.

### Baseline characteristics of patients

Demographic factors included age (numeric), sex, and race (categorized as white vs none-white). Other patient specific clinical variables included: Body mass index (categorized as 20–30 BMI, < 20 BMI, 30–40 BMI, > 40 BMI), alcohol abuse, current smoker, steroid use for a chronic condition, dyspnoea (none, on exertion, at rest), functional health status (independent, partially dependent, totally dependent), American Society of Anesthesiologists physical status (ASA) (1 & 2 combined, 3, 4, or 5), stroke with neurological deficit, COPD, ongoing pneumonia, myocardial infarction 6 months prior to surgery, previous PCI, previous cardiac surgery, congestive heart failure (CHF) in 30 days prior to surgery, peripheral vascular disease (PVD), currently on dialysis, acute renal failure, diabetes mellitus (none, NIDDM, or IDDM), sepsis status (none, systemic inflammatory response syndrome (SIRS)/sepsis, or septic shock), disseminated cancer, radiotherapy for malignancy in last 90 days, chemotherapy for malignancy in 30 days prior to surgery, bleeding disorder, surgical specialty (orthopaedic, vascular, gynaecological & urological, ENT & plastic, thoracic, or neurosurgical), year of operation (2008–2012), emergency (emergent/urgent or elective) cases and anaesthesia technique (general or none-general anaesthesia). Further details on the definition of those factors can be found under: https://www.facs.org/quality-programs/acs-nsqip/program-specifics/participant-use.

### Outcomes

Outcomes of interest were intraoperative CPR, CPR within 30 days postopertively, and mortality within 30 days after the operation. Intraoperative CPR was extracted from the NSQIP variable "type of intraoperative occurrence".

### Statistical analysis

Univariate analysis was performed to compare demographics and patient specific clinical factors between the CPR and none-CPR group (intra- and 30-day postoperatively), as wells as death within 30 days using Pearson Chi-square tests for all categorical variables and unpaired t-test for all discrete numerical variables.

Model fitting with logistic regression for prediction of outcome is very sensitive to collinearities of independent variables. Therefore, multicollinearity was tested using variation inflation factors (VIF) and tolerance (TOL).[4] Due to large set of predictors, forward stepwise logistic regression was performed to identify those independent variables with a significant association with intraoperative, postoperative CPR, as well as 30-day mortality. The significance level of the score Chi-square included in the model was set at 0.15, the level of the Wald Chi-square to stay in the model was set at 0.05.

Conventional multivariable logistic regression was not considered appropriate to identify associations between independent and dependent variables in this study because of the rarity of CPR, which would cause sample bias. The degree of bias depends on the number of cases in the less frequent of the two groups. This could lead to a critical underestimation of the probability of occurrence.[5]

Firth described that bias can be corrected during the maximization procedure by applying Jeffrey’s invariant prior to the logistic likelihood and applying the maximum posterior estimate. Firth`s penalized likelihood is a general approach to minimize small-sample bias in maximum likelihood estimation. When applying logistic regression, penalized likelihood also has the benefit of generating finite, consistent estimates of regression parameters when the maximum likelihood estimates do not even exist because of complete or quasi-complete separation.[5, 6] We used the logistf package from R (https://www.r-project.org) to run the Firth’s bias-reduced logistic regression.[7] For comparing the goodness of fit of our models we performed a penalized likelihood ratio test.

The C-statistics (ROC curves as supplement 1) and Somers’ Dxy were calculated with R’s Hmisc package (https://www.r-project.org) and the somers2 function to evaluate the appropriateness of the models and to choose the best model. [8] A priori, intraoperative and postoperative CPR were intended to be added as independent variables for 30 day-mortality and intraoperative CPR for postoperative CPR prediction.

Sensitivity and specificity were calculated with cut-off values to produce the best-balanced combination of both. We also calculated the sensitivity and the specificity for every outcome using the five factors with the strongest association, as well as for ASA physical status only. This was carried out to compare how accurate a prediction would be without using all variables with known significance to possibly receive a more practical simplified prediction model for everyday clinical management.

A prediction model for CPR and mortality does not have a clinical or scientific significance without assessing the validity of the results, hence we subdivided our data beforehand into a calibration and a validation dataset. Stratified random sampling was performed with SPSS (IBM^™^, Armonk, New York) to produce a calibration dataset containing 80% of the events and none-events and the validation set containing the remaining 20%.

## Results

**Fig 1:**
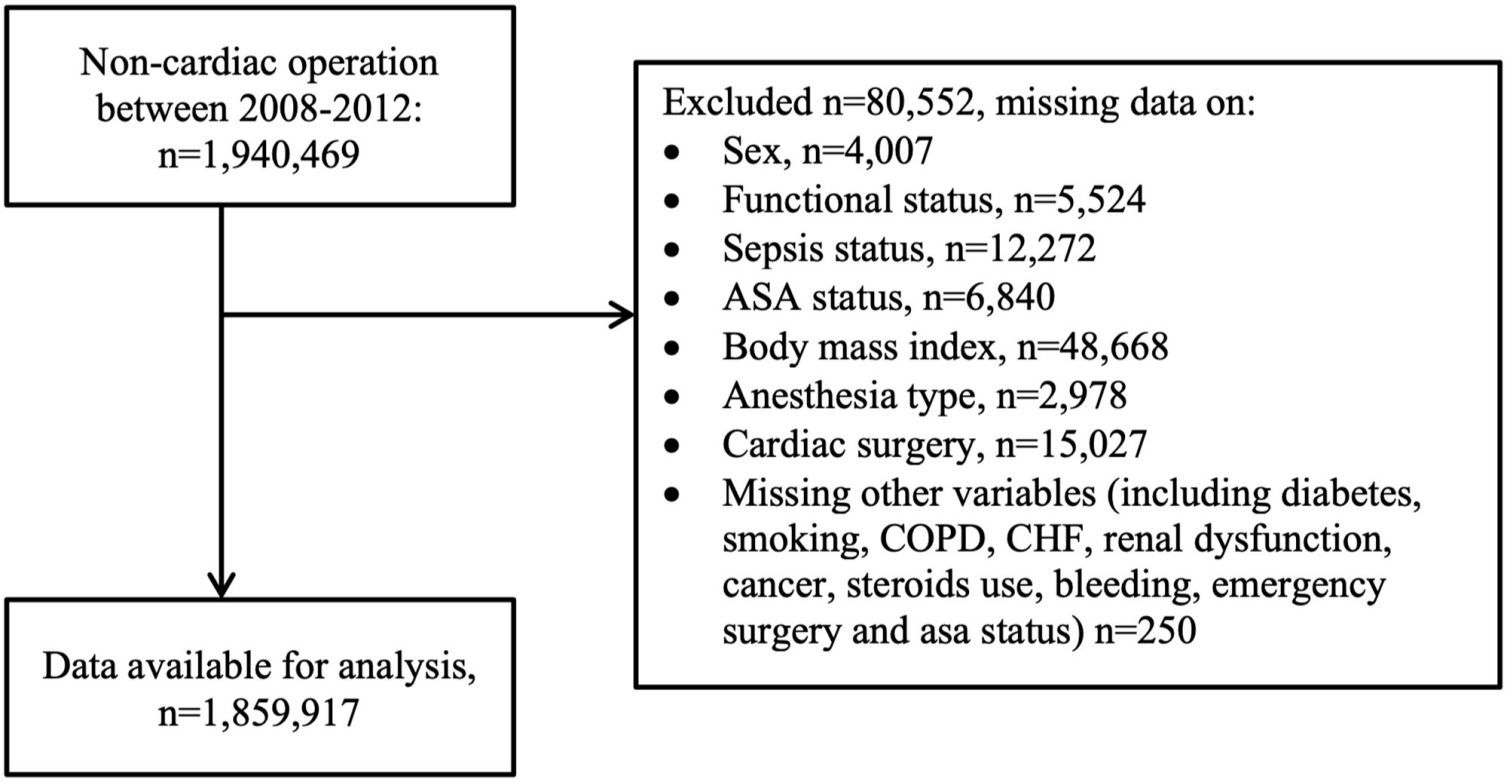
Flow diagram of patient selection. COPD: Chronic Obstructive Pulmonary Disease, CHF: Congestive Heart Failure, ASA: American Society of Anesthesiologists.

At baseline the ACS-NSQIP dataset contained 1,940,469 patients with non-cardiac surgical interventions. After removing 80,552 patients (4.15%) due to missing values, at random, primarily in sex, functional status, BMI, sepsis information or type of anaesthesia (other missing values n < 100 per variable), n = 1,859,917 remained for the analysis (Fig 1). There were 560 intraoperative CPR events (incidence rate of 0.03%), 6183 postoperative CPR events (incidence rate of 0.33%) and 23,265 deaths (mortality rate 1.25%) in the first 30 postoperative days.

### Patients characteristics and univariate analysis

The incidence of intraoperative CPR, postoperative CPR and mortality decreased between 2008 and 2012 (Table 1). The differences between groups of CPR and mortality at 30 days were all highly significant (p< .001) before adjusting for patient specific factors. Unadjusted, patients who had to be resuscitated or died within 30 days postoperatively tended to be older, male, smokers, and of poorer functional status. Comorbidities were more likely to be present in the CPR resuscitated and the deceased groups, which is reflected in the higher ASA physical status of patients who made up these groups. Specifically, the intraoperative CPR group shows a high percentage of patients with a history of PCI, previous cardiac surgery and PVD. More than 20% of patients in the perioperative CPR or mortality groups had a known bleeding disorder according to the NSQIP definition (due to either a deficiency of blood clotting elements or due to anticoagulation therapy excluding aspirin, that was not discontinued prior to surgery), in comparison to 5% of patients who were not require CPR and survived. Fifty percent of the intraoperative CPR events were during urgent/emergent procedures, whereas 35% of postoperative CPR events were after urgent/emergent interventions. Patients who did not survive during the first 30 postoperative days had urgent/emergent surgery in 46% of cases. Regarding surgical specialties; vascular and thoracic surgery had the highest incidence of intraoperative (0.13% and 0.06%) and postoperative CPR (0.91% and 0.87%), as well as mortality (2.71% and 2.97%), respectively (Table 2).

**Table 1:** Patient characteristics in the intraoperative CPR, postoperative CPR and 30d-Mortality.

		Intraop CPR		Postop CPR		At 30 days	
PREDICTOR	Level	No	Yes	No	Yes	alive	dead
AGE*		56.2±16.8	67.2±14.5	56.2±16.8	68.3±13.5	56±16.7	71.7±13.6
**YEAR OF OPERATION [IN % PER YEAR]**							
	2008	99.95	0.05	99.60	0.40	98.41	1.59
	2009	99.95	0.05	99.62	0.38	98.51	1.49
	2010	99.96	0.04	99.66	0.34	98.70	1.30
	2011	99.98	0.02	99.67	0.33	98.80	1.20
	2012	99.99	0.01	99.73	0.27	99.06	0.94
**GENDER**	female	57.6	41.8	57.7	42.3	57.7	47.9
**DIABETES**	no DM	84.9	72.5	84.9	67.6	85.0	73.0
	NIDDM	9.3	13.9	9.3	13.5	9.3	11.9
	IDDM	5.8	13.6	5.7	18.9	5.7	15.1
**SMOKER**	no smoker	80.7	75.0	80.7	76.7	80.7	78.6
	smoker	19.3	25.0	19.3	23.3	19.3	21.4
**ALCOHOLIC DRINKS/DAY >2**							
	no alcohol	98.2	95.7	98.2	97.2	98.2	96.9
	alcohol	1.8	4.3	1.8	2.8	1.8	3.1
**DYSPNEA**	no dyspnea	91.3	71.1	91.4	73.9	91.6	70.7
	dyspnea on exertion	7.7	14.8	7.7	16.8	7.6	16.1
	dyspnea at rest	1.0	14.1	0.9	10.3	0.8	13.2
**FUNCTIONAL HEALTH STATUS**							
	Independent	95.3	64.6	95.4	68.7	95.8	57.2
	Partially dependent	3.6	14.3	3.5	17.2	3.3	21.7
	Totally dependent	1.1	21.1	1.1	14.1	0.8	21.1
**COPD**	No	95.3	84.3	95.3	82.5	95.5	80.5
	Yes	4.7	15.7	4.7	17.5	4.5	19.5
**PNEUMONIA**	No	99.7	94.5	99.7	96.3	99.7	94.1
	Yes	0.3	5.5	0.3	3.7	0.3	5.9
**CHF**	No	99.3	90.0	99.3	91.5	99.4	91.0
	Yes	0.7	10.0	0.7	8.5	0.6	9.0
**MI LAST 6 MONTHS**	no MI	99.6	94.3	99.6	96.1	99.7	96.1
	MI	0.4	5.7	0.4	3.9	0.3	3.9
**PREVIOUS PCI**	no PCI	96.3	83.0	96.3	89.0	96.4	90.2
	PCI	3.7	17.0	3.7	11.0	3.6	9.8
**PREVIOUS CARDIAC SURGERY**							
	no cardiac surgery	96.3	80.0	99.7	86.3	96.4	87.7
	cardiac surgery	3.7	20.0	3.6	13.7	3.6	12.3
**PVD**	no PVD	97.4	85.7	97.4	88.9	97.5	90.9
	PVD	2.6	14.3	2.6	11.1	2.5	9.1
**ACUTE RENAL FAILURE**	No	99.5	93.9	99.6	94.2	99.6	93.2
	Yes	0.5	6.1	0.4	5.8	0.4	6.8
**ON DIALYSIS**	No	98.4	90.2	98.4	86.5	98.5	88.9
	Yes	1.6	9.8	1.6	13.5	1.5	11.1
**STROKE WITH NEUROLOGICAL DEFICIT**							
	No	98.5	94.1	98.5	94.4	98.6	93.3
	Yes	1.5	5.9	1.5	5.6	1.4	6.7
**DISSEMINATED CANCER**	No	98.0	95.0	98.0	95.0	98.1	87.6
	Yes	2.0	5.0	2.0	5.0	1.9	12.4
**CORTICOSTEROID USE**	No	96.9	92.7	96.9	91.7	97.0	89.2
	Yes	3.1	7.3	3.1	8.3	3.0	10.8
**BLEEDING DISORDER**	No	95.0	78.6	95.0	79.2	95.2	76.7
	Yes	5.0	21.4	5.0	20.8	4.8	23.3
**CHEMOTHERAPY LAST 30 DAYS**							
	No	99.0	97.1	99.0	98.3	99.1	96.2
	Yes	1.0	2.9	1.0	1.7	0.9	3.8
**RADIOTHERAPY LAST 90 DAYS**							
	No	99.5	98.9	99.5	99.0	99.5	98.4
	Yes	0.5	1.1	0.5	1.0	0.5	1.6
**EMERGENCY CASE**	Elective	89.5	49.6	89.6	64.4	90.0	53.6
	Emergent	10.5	50.4	10.4	35.6	10.0	46.4
**SIRS OR SEPSIS**	None	93.5	62.1	93.5	64.8	93.9	54.5
	SIRS or sepsis	6.0	20.9	5.9	24.0	5.7	29.2
	Septic shock	9.6	17.0	0.5	11.2	0.4	16.3
**ASA**	ASA 1 & 2	55.5	7.3	55.6	8.4	56.1	4.8
	ASA 3	38.6	28.9	38.5	46.1	38.5	39.7
	ASA 4	5.7	41.3	5.6	39.6	5.2	47.7
	ASA 5	0.2	22.5	0.2	5.8	0.1	7.9
**BMI**	20–30 BMI	54.6	57.1	54.6	56.6	54.6	58.1
	< 20 BMI	4.9	9.8	4.9	10.0	4.8	13.7
	30–40 BMI	29.7	26.6	29.7	24.3	29.8	21.5
	> 40 BMI	10.8	6.4	10.8	9.1	10.8	6.7
**RACE**	white	85.3	79.6	85.4	77.3	85.4	84.7
	other	14.7	20.4	14.6	22.7	14.6	15.3
**SURGICAL SPECIALTIES**	General	60.7	47.5	60.7	59.2	60.6	64.3
	Orthopedic	11.9	3.4	11.9	5.5	12.0	6.0
	vascular	9.6	42.5	9.6	26.3	9.5	20.9
	GYN & Urology	9.4	2.3	9.4	2.9	9.5	2.3
	neurosurgery	3.1	1.1	3.1	2.4	3.1	3.3
	ENT & plastic	4.2	1.1	4.2	1.0	4.3	0.8
	Thoracic	1.0	2.1	1.0	2.7	1.9	2.4
**ANESTHESIA TECHNIQUE**							
	General	91.0	98.0	91.0	94.6	90.9	94.6
	Other	9.0	2.0	9.0	5.4	9.1	5.4

Except for age (means ±SD), data are presented as percent per level. $ Differences between groups are all highly significant (< 0.0001); DM = diabetes mellitus, NIDDM = none-insulin dependent diabetes mellitus, IDDM = insulin dependent diabetes mellitus, EtOH = alcohol, COPD = chronic obstructive pulmonary disease, CHF = congestive heart failure, MI = myocardial infarction, PCI = percutaneous coronary intervention, PVD = peripheral vascular disease, SIRS = systemic inflammatory response syndrome, ASA = American Society of Anesthesiologists physical status classification system, BMI = body mass index, GYN = gynecological surgery, ENT = ear, nose & throat surgery.

**Table 2:** Firth’s penalized-likelihood logistic regression for perioperative CPR and 30d-Mortality.

		Intraoperative CPR		Postoeprative CPR		30-day Mortality	
Predictor	level	*OR (95% CI)*	*p*	*OR (95% CI)*	*p*	*OR (95% CI)*	*p*
**Age**		1.0 (1.0–1.0)	0.11	1.0 (1.0–1.0)	<0.01	1.1 (1.0–1.1)	<0.01
**Gender**	**male**	1.2 (1.0–1.4)	0.11	1.4 (1.3–1.5)	<0.01	1.1 (1.1–1.1)	<0.01
**BMI**	**20–30 BMI**						
	**< 20 BMI**	1.2 (0.9–1.6)	0.26	1.3 (1.2–1.4)	<0.01	1.7 (1.6–1.8)	<0.01
	**30–40 BMI**	0.9 (0.7–1.1)	0.32	0.9 (0.8–0.9)	<0.01	0.8 (0.8–0.9)	<0.01
	**> 40 BMI**	0.7 (0.4–1.0)	0.04	0.9 (0.8–1.0)	0.17	0.8 (0.8–0.9)	<0.01
**EtOH > 2 drinks/day**	**Yes**					1.2 (1.1–1.3)	<0.01
**Smoker**	**Yes**			1.1 (1.0–1.2)	<0.01	1.2 (1.1–1.2)	<0.01
**Corticosteroids**	**Yes**			1.2 (1.1–1.3)	<0.01	1.5 (1.4–1.5)	<0.01
**Dyspnea**	**on exertion**	1.0 (0.8–1.4)	0.75	1.3 (1.2–1.4)	<0.01	1.2 (1.2–1.3)	<0.01
	**dyspnea at rest**	1.4 (1.0–2.0)	0.03	1.5 (1.3–1.7)	<0.01	1.6 (1.5–1.8)	<0.01
**Functional status**	**independent**						
	**partially dependent**			1.5 (1.4–1.6)	<0.01	2.1 (2.0–2.2)	<0.01
	**totally dependent**			1.7 (1.5–1.9)	<0.01	3.3 (3.1–3.5)	<0.01
**ASA Physical Status**	**ASA 1 & 2**						
	**ASA 3**	3.7 (2.5–5.6)	<0.01	4.3 (3.9–4.8)	<0.01	4.1 (3.8–4.4)	<0.01
	**ASA 4**	16.9 (11.2–25.9)	<0.01	9.2 (8.1–10.4)	<0.01	11.0 (10.1–11.9)	<0.01
	**ASA 5**	139.9 (87.0–228.2)	<0.01	17.6 (14.6–21.1)	<0.01	31.6 (27.9–35.7)	<0.01
**Stroke with deficit**	**Yes**					1.2 (1.1–1.3)	<0.01
**COPD**	**Yes**			1.2 (1.1–1.2)	<0.01	1.2 (1.2–1.3)	<0.01
**Pneumonia**	**Yes**			0.9 (0.7–1.0)	0.05	1.2 (1.1–1.3)	<0.01
**MI last 6 months**	**Yes**			1.3 (1.1–1.5)	<0.01		
**Previous PCI**	**Yes**	1.5 (1.1–1.9)	0.01			1.0 (0.9–1.0)	0.15
**Previous cardiac surgery**	**Yes**	1.4 (1.1–1.7)	0.02	1.1 (1.0–1.2)	0.02		
**CHF last 30 days**	**Yes**	1.2 (0.8–1.6)	0.43	1.6 (1.4–1.7)	0.00	1.4 (1.3–1.5)	<0.01
**PVD**	**Yes**			1.2 (1.1–1.3)	<0.01		
**On dialysis**	**Yes**			2.0 (1.8–2.2)	<0.01	1.6 (1.5–1.8)	<0.01
**Acute renal failure**	**Yes**			1.2 (1.1–1.4)	0.01	1.4 (1.3–1.6)	<0.01
**Diabetes**	**NIDDM**			1.1 (1.0–1.2)	0.15	0.9 (0.9–0.9)	<0.01
	**IDDM**			1.4 (1.3–1.5)	<0.01	1.0 (1.0–1.1)	0.17
**SIRS or Sepsis**	**SIRS or sepsis**	1.2 (0.9–1.6)	0.14	2.1 (1.9–2.2)	<0.01	2.3 (2.2–2.5)	<0.01
	**septic shock**	1.9 (1.4–2.7)	<0.01	2.6 (2.3–3.0)	<0.01	4.3 (4.0–4.7)	<0.01
**Disseminated cancer**	**Yes**	1.8 (1.2–2.7)	0.01	1.3 (1.1–1.5)	<0.01	5.0 (4.7–5.3)	<0.01
**Radiotherapy last 90 days**	**Yes**					1.2 (1.0–1.4)	0.02
**Chemotherapy last 30 days**	**Yes**					1.4 (1.3–1.6)	<0.01
**Bleeding disorders**	**Yes**			1.1 (1.0–1.2)	0.01	1.3 (1.2–1.3)	<0.01
**Surgical Specialty**	**general surgery**						
	**orthopedics**	1.0 (0.6–1.7)	0.86	0.7 (0.6–0.8)	<0.01	0.7 (0.7–0.8)	<0.01
	**vascular**	2.7 (2.2–3.4)	0.00	1.1 (1.1–1.2)	<0.01	0.8 (0.8–0.9)	<0.01
	**gyn & urology**	1.0 (0.5–1.7)	0.95	0.6 (0.5–0.7)	<0.01	0.6 (0.5–0.6)	<0.01
	**neurosurgery**	0.6 (0.2–1.3)	0.21	0.9 (0.7–1.1)	0.24	1.2 (1.1–1.3)	<0.01
	**ENT & plastic surgery**	0.8 (0.3–1.9)	0.69	0.5 (0.4–0.7)	<0.01	0.4 (0.4–0.5)	<0.01
	**thoracic**	1.9 (0.9–3.5)	0.11	1.6 (1.3–1.9)	<0.01	1.2 (1.1–1.4)	<0.01
**Year of Operation**	**2008**						
	**2009**	1.1 (0.8–1.4)	0.50				
	**2010**	1.0 (0.7–1.3)	0.90				
	**2011**	0.5 (0.4–0.7)	<0.01				
	**2012**	0.4 (0.3–0.5)	<0.01				
	**not emergent**						
**Emergency case**	**Yes**	2.4 (1.8–3.0)	<0.01	1.7 (1.6–1.9)	<0.01	2.1 (2.0–2.2)	<0.01
**Anesthesia technique**	**Not General**	0.2 (0.1–0.4)	<0.01	0.6 (0.5–0.7)	<0.01	0.7 (0.6–0.7)	<0.01
**Intraoperative CPR**	**Yes**			2.7 (1.8–3.8)	<0.01	19.1 (14.6–24.8)	<0.01
**Postoperative CPR**	**Yes**					118.2 (109.3–127.8)	<0.01

BMI = Body Mass Index, ASA = American Society of Anesthesiologists physical status classification system, COPD = chronic obstructive pulmonary disease, MI = myocardial infarction,

PCI = percutaneous coronary intervention, CHF = congestive heart failure, PVD = peripheral vascular disease, ARF = Acute Renal Failure, NIDDM = none-insulin dependent diabetes mellitus,

IDDM = insulin dependent diabetes mellitus, SIRS = systemic inflammatory response syndrome, GYN = gynocological surgery, ENT = ear, nose & throat surgery,

CPR = Cardiopulmonary Resuscitation. OR = Odds Ratio, na = not available, hs = highly significant (< 0.0001)

### Stepwise logistic regression

The multicollinearity tests did not show any highly correlated independent variables before the stepwise logistic regression was performed. For further analysis 14 of 29 potential predictors remained for intraoperative CPR, 23 of 29 for postoperative CPR, and 25 of 29 for 30-day mortality.

### Firth’s penalized-likelihood logistic regression

In addition to the aforementioned potential predictors, we determined a priori to include intraoperative CPR as an independent variable for postoperative CPR and mortality, as well as postoperative CPR for 30-day mortality for Firth’s penalized-likelihood logistic regression (Table 2).

**Fig 2:**
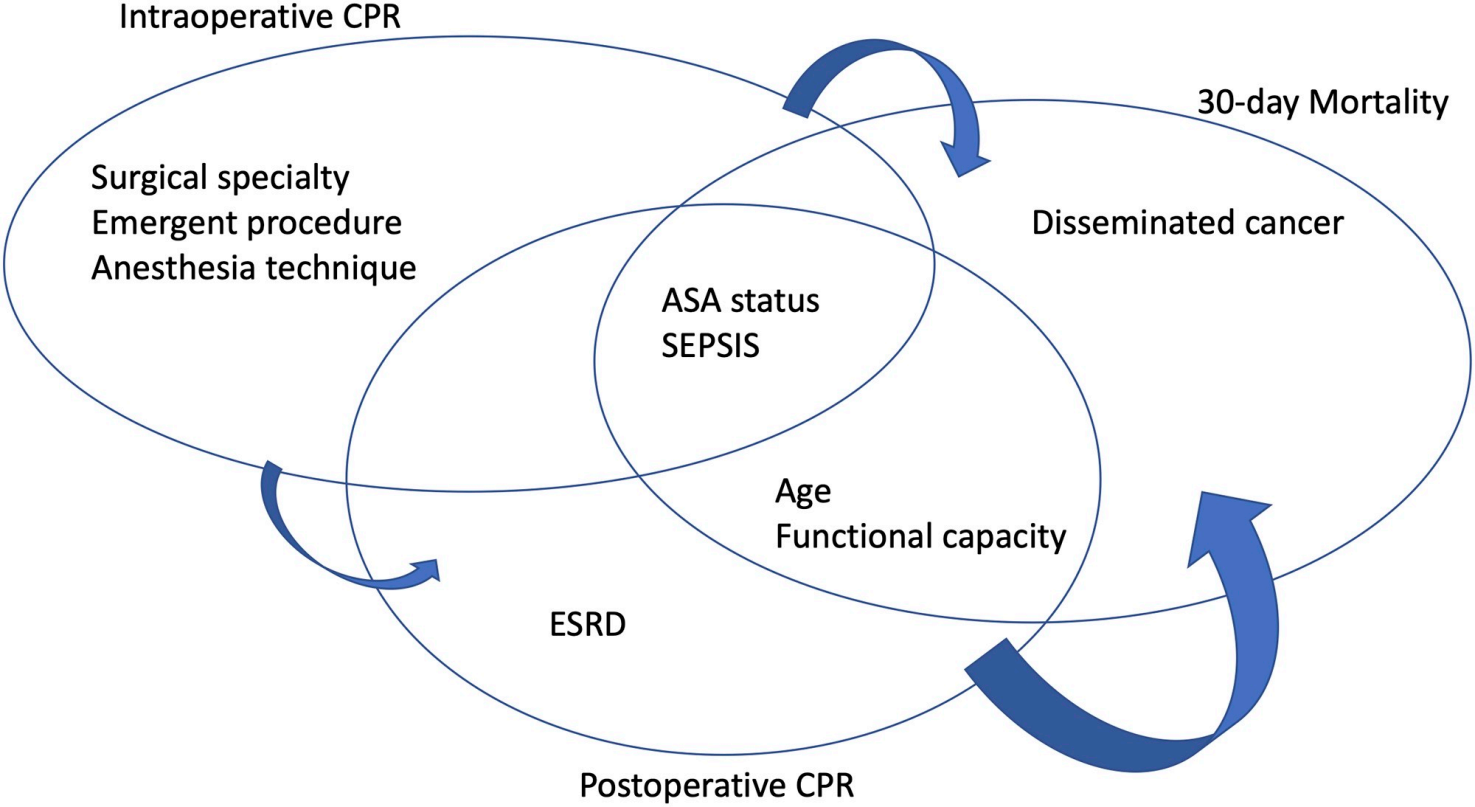
Schematic summary of the 5 main factors for prediction of intraoperative CPR, postoperative CPR and 30-day mortality.

The five strongest predictors of intraoperative CPR were ASA physical status (OR = 3.7, 17 and 140 for ASA 3, 4 and 5 respectively), SIRS/sepsis (OR up to 1.9 for septic shock), surgical procedure (OR = 2.7 for vascular surgery, and OR = 1.9 for thoracic surgery), urgent/emergency case (OR = 2.4) and anaesthesia technique (OR = 0.24 for none-general anaesthesia). Aside from intraoperative CPR with an OR of 2.7, ASA physical status (OR = 4.3, 9 and 18 for ASA 3, 4 and 5, respectively), age (OR = 1.02/patient year), functional status (OR = 1.5 and 1.7 for partially and totally dependent), end stage renal disease on dialysis (OR = 2.0), SIRS/sepsis variable (OR = 2.1 and 2.6 for SIRS/sepsis and septic shock, respectively) were the most significant predictors for the occurrence of postoperative CPR (Fig 2).

The most significant independent variables (by odd ratio and confidence intervals) for the prediction of 30-day mortality were ASA physical status (OR of 4.1, 11 and 32 for ASA 3, 4 and 5, respectively), age (OR of 1.05/patient year), functional status (OR of 2.1 and 3.3 for partially and totally dependent), SIRS/sepsis (OR of 2.4 and 4.3 for SIRS or sepsis versus septic shock) and disseminated cancer (OR of 5). As to be expected, the a priori determined variables intraoperative and postoperative CPR were strongly associated with postoperative death (OR = 19 intraoperative CPR versus postoperative CPR = 118), Table 2.

### Model appropriateness

As Table 3 shows, the C-statistics for the full models, the reduced five-factor models, and the ASA physical status models all revealed good prediction quality for the different models, whereas Somers’ correlations decreased with the reduction of the number of independent variables in the different models. This finding is reflected in the sensitivity and specificity analysis presented in Table 4, which lists the different models and the corresponding course of sensitivity and specificity with reduction of independent variables. We deduce that our predictions based on the ASA physical status only was less predictive, in contrast to our full models or five-factor model approach. ASA classification alone could be a useful crude estimate for the overall risk.

**Table 3:** C-statistics & Somer’s rank correlations.

		FULL MODEL	5 MAIN FACTORS	ASA ONLY
**INTAOPERATIVE CPR**	***Calibration***			
	C-statistic	0.91	0.89	0.86
	Somers’ correlation	0.81	0.77	0.71
	***Validation***			
	C-statistic	0.92	0.91	0.88
	Somers’ correlation	0.84	0.82	0.76
**POSTOPERATIVE CPR**	***Calibration***			
	C-statistic	0.88	0.87	0.81
	Somers’ correlation	0.76	0.73	0.62
	***Validation***			
	C-statistic	0.88	0.86	0.81
	Somers’ correlation	0.76	0.72	0.62
**30D MORTALITY**	***Calibration***			
	C-statistic	0.95	0.92	0.85
	Somers’ correlation	0.90	0.84	0.71
	***Validation***			
	C-statistic	0.95	0.92	0.86
	Somers’ correlation	0.90	0.85	0.71

Full Model: All predictors that showed significant influence in primary stepwise regression.

5 main factors for intraoperative CPR: ASA physical status, SISRS or sepsis, surgical specialty, emergency case, anesthesia technique

5 main factors for postoperative CPR: age, functional status, ASA physical status, dialysis, sirs or sepsis

5 main factors for 30d mortality: age, functional status, ASA physical status, SISRS or sepsis, disseminated cancer

**Table 4:** Calibration & validation of Firth’s logistic regression model of intraoperative CPR, postoperative CPR and 30d-Mortality.

Calibration									
	Intraop CPR			Postop CPR			Mortality		
Statistics	full model	5 main factors	ASA only	full model	5 main factors	ASA only	full model	5 main factors	ASA only
**specificity**	0.84	0.88	0.94	0.79	0.80	0.56	0.89	0.86	0.56
**sensitivity**	0.83	0.74	0.63	0.81	0.77	0.92	0.87	0.83	0.95
**Validation**									
**specificity**	0.86	0.85	0.94	0.79	0.78	0.56	0.89	0.84	0.56
**sensitivity**	0.88	0.87	0.67	0.81	0.77	0.91	0.87	0.84	0.95

Full Model: Include all predictors that showed significant association in the full stepwise regression.

Five-factor models:

5 main factors for intraoperative CPR: ASA physical status, SISRS or sepsis, surgical specialty, emergency case, anesthesia technique.

5 main factors for postoperative CPR: Age, functional status, ASA physical status, dialysis, SIRS or sepsis.

5 main factors for 30d mortality: Age, functional status, ASA physical status, SISRS or sepsis, disseminated cancer.

The validity of the model was checked by internal validation with the separation of our data beforehand into a calibration and a validation dataset containing 80% and 20% of the data, respectively. The resulting sensitivities and specificities for all models performed in the two datasets are almost identical and are presented in detail in Table 4.

The differences regarding validity of the models–full, five-factor model and ASA only–is best graphically represented with ROC curves of the calibration data and are available online as S1–S3 Figs.

## Discussion

In this study we presented the incidence course overtime for intraoperative CPR, postoperative CPR and 30-day mortality. We identified risk factors, 14 statistically significant risk factors for intraoperative CPR, 23 for postoperative CPR, and 25 for 30-day mortality. and We were able to predict perioperative cardiac arrest with a high sensitivity and specificity using a simplified model of five main risk factors to predict intraoperative and postoperative CPR as well as 30-day mortality. We believe that our five-factor model is simple and might be superior to the NSQIP surgical risk calculator in a busy, everyday clinical practice. Furthermore, we demonstrated that the five risk factors having the highest predictive value for each clinical endpoint are not constant among each of these three endpoints. While the ASA status and SIRS/sepsis status were uniformly important for predicting all three endpoints, other contributing risk factors differed. Important risk factors for both intraoperative CPR and 30-day mortality included the functional status and age. However, other factors existed that were predictive of postoperative CPR, and included intraoperative CPR and the need for dialysis. Additionally, postoperative CPR and disseminated cancer were identified to contribute to 30-day mortality.

The first goal of our study was to report the incidence of intraoperative CPR, postoperative CPR, and 30-day mortality within the ACS-NSQIP data overtime. We showed an overall intraoperative and postoperative CPR incidence of 0.03% and 0.033%, respectively. Our calculated incidence of overall mortality is 1.25%. The overall rate of CPR as well as 30-day mortality declined over time during the study period from 2008 to 2012 (Table 2). Compared to other studies during earlier periods, the rate of perioperative CPR and mortality according to the NSQIP database appears to have decreased.[2, 9] However, the data on intraoperative CPR was not recorded within the database after 2012 limiting us of pursuing the trend during the subsequent years.

A large retrospective analysis [10] and a single-centre experience published in 2014 demonstrated an overall incidence of cardiac arrest of 7/10,000 patients within 24 hours of surgery.[11] The cause for the reduction in CPR rates over time cannot be derived from our data, however this is most likely multifactorial. Our study demonstrated that a high ASA status and a patient meeting SIRS/sepsis criterion are both major risk factors for perioperative CPR and death. Therefore, there is clearly a need for medical optimization prior to an elective surgery.

The second goal of our study was to identify specific risk-factors for intra- and postoperative need for CPR, and 30-day mortality and to create a simplified model to predict the event occurrence. Kazaure et al. previously analysed the incidence of perioperative CPR within the ACS-NSQIP dataset.[2] Kazaure identified age, a higher ASA physical status, and disseminated cancer as predictors for perioperative CPR and 30-day mortality. However, their group identified other variables, such as COPD (OR 1.22), which were not among our five strongest predictors while some of our most significant risk factors, such as emergency case, have not been considered in their analysis.

Some studies that have looked at perioperative mortality related to cardiac arrest requiring CPR, focusing on anaesthesia related risk factors. For example, Nunnaly et al. analysed 1.69 million data sets from the National Anaesthesia Clinical Outcomes Registry and identified age and high ASA physical status as independent risk factors for anaesthesia related perioperative death, but their group did not account for surgical risk.[12] Hohn, et al. differentiated between anaesthesia-related and anaesthesia-contributed CPR within the first 24h after surgery.[13] They identified emergency cases, ASA physical status and pre-existing cardiomyopathy as main risk factors. Ellis, et al. ^11^ further characterized the cause of perioperative CPR by differentiating between anaesthesia-attributable or anaesthesia-contributory CPR. Their data showed that 23% of all cardiac arrests were anaesthesia-related.[11]

A well-established and accurate predictor of postop morbidity and mortality is the ACS-NSQIP risk calculator method. In fact, this calculator was recently adopted by the 2014 ACC/AHA guideline as a means to estimate the likelihood of perioperative major adverse cardiac events.[14] While this calculator is useful, it is complex, time consuming, and requires very detailed patient information (https://Riskcalculator.Facs.org). For this reason, our estimation could be a very useful quick tool to estimate risk and to prompt clinicians to increase monitoring and apply appropriate intervention.

Regional anaesthesia or monitored anaesthesia care (MAC) resulted in less cardiac arrest requiring CPR in this study. Other retrospective studies have shown similar favourable outcomes with regional compared to general anaesthesia.[15, 16] This may need to be taken into consideration when caring for high risk patients.

Finally, we demonstrated that survival after intraoperative CPR is six times higher than survival after postoperative CPR. This observation has been shown in other studies, [17] and could be related to the fact that intraoperative cardiac arrest is witnessed, with known likely culprit and availability of resources for immediate treatment.

This study has some limitations. There is no data on type of event that led to CPR (cardiac, respiratory or others). Unobserved and hidden factors could still potentially bias the results. For example, data related to medical centre factors were not available and could not be included in the model. Also, the conclusion related to type of anaesthesia could have been biased, as regional anaesthesia is not feasible for some procedures. Specific definition of variables in the NSQIP might lead to different incidence rate.

Lastly, excluding cardiac arrest witnessed on the day of surgery, the majority of 30-day mortality occurred without someone performing CPR on the patient. This could be caused by a variety of factors, including unwitnessed death after discharge or patient’s decision to activate DNR order or to proceed with comfort-focused care.

## Conclusions

In conclusion, we demonstrated a decrease in the incidence of cardiac arrest requiring CPR over time. We identified different risk factors for intraoperative CPR, postoperative CPR and perioperative mortality. We were able to predict these events with a high sensitivity and specificity using a simplified, five-factor models. This prediction might be helpful to identify patients at risk early and to guide clinical practice accordingly.

## Supporting information

S1 Fig ROC curve of predicting intraoperative CPR (Calibration Model); solid line = full model, dashed line = five-factor model, dotted line = ASA only model. (TIFF)

S2 Fig ROC curve of predicting postoperative CPR (Calibration Model); solid line = full model, dashed line = five-factor model, dotted line = ASA only model. (TIFF)

S3 Fig ROC curve of predicting postoperative CPR (Calibration Model); solid line = full model, dashed line = five-factor model, dotted line = ASA only model. (TIFF)
