# Supplementary figures and images for: Incidence and prediction of intraoperative and postoperative cardiac arrest requiring cardiopulmonary resuscitation and 30-day mortality in non-cardiac surgical patients
Source: PLoS One. 2020 Jan 22;15(1):e0225939. doi: 10.1371/journal.pone.0225939 (PMC6975552; doi:10.1371/journal.pone.0225939)

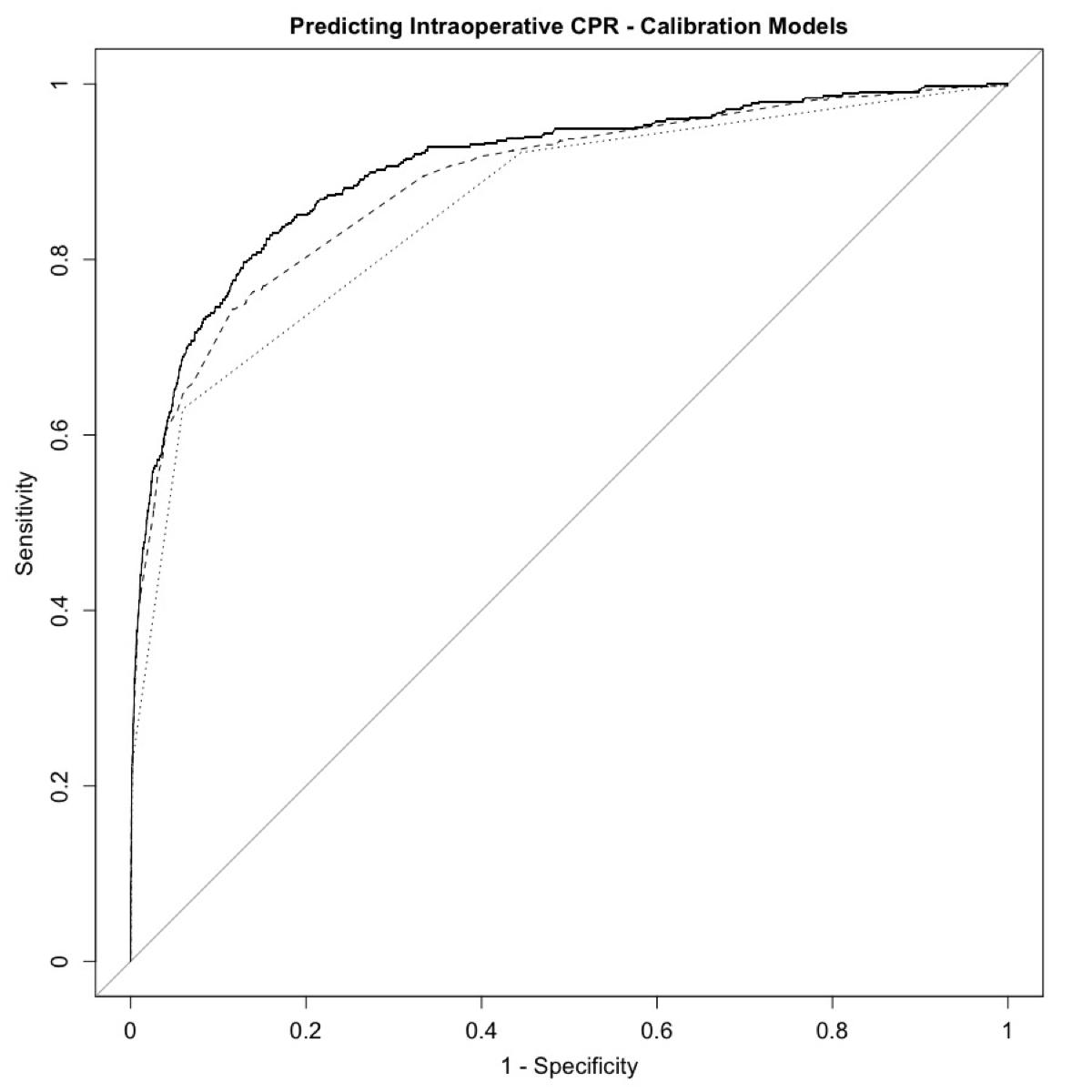

Supplement: S1 Fig — (TIFF) [file pone.0225939.s001.tiff]

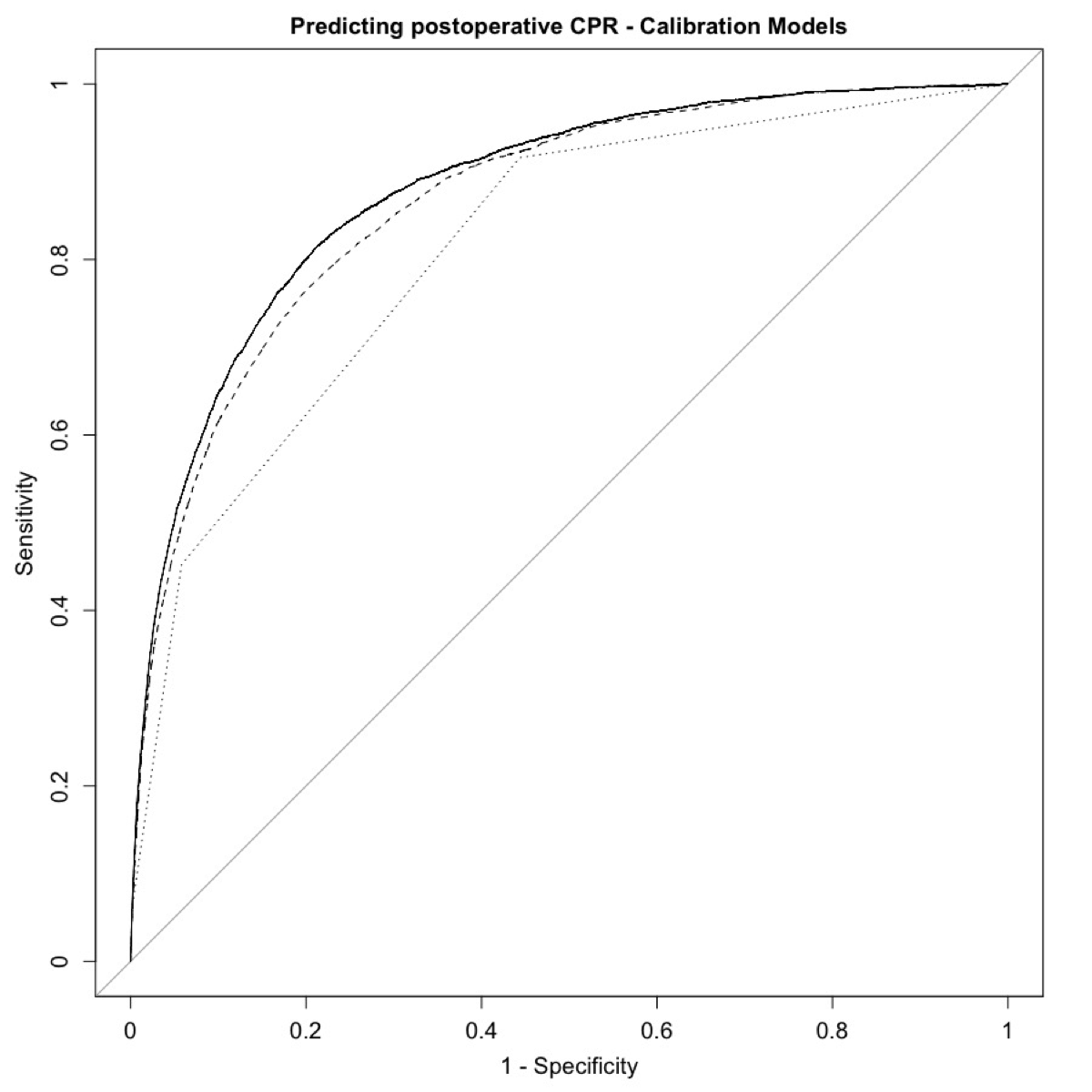

Supplement: S2 Fig — (TIFF) [file pone.0225939.s002.tiff]

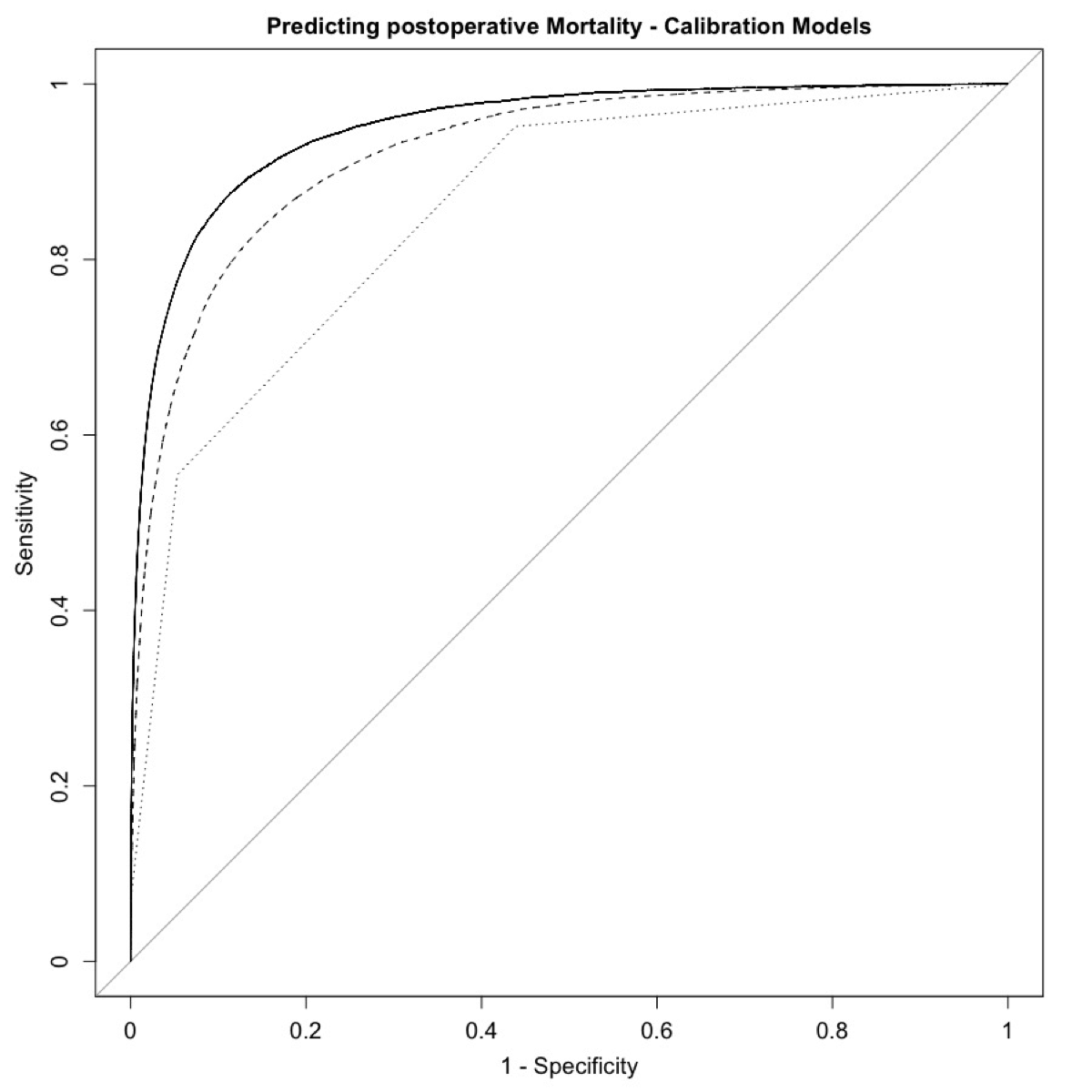

Supplement: S3 Fig — (TIFF) [file pone.0225939.s003.tiff]
